# The Influence of Genes on the “Killer Plasmid” of *Dinoroseobacter shibae* on Its Symbiosis With the Dinoflagellate *Prorocentrum minimum*

**DOI:** 10.3389/fmicb.2021.804767

**Published:** 2022-01-28

**Authors:** Johannes Mansky, Hui Wang, Matthias Ebert, Elisabeth Härtig, Dieter Jahn, Jürgen Tomasch, Irene Wagner-Döbler

**Affiliations:** ^1^Institute for Microbiology, Technical University of Braunschweig, Braunschweig, Germany; ^2^Laboratory of Anoxygenic Phototrophs, Institute of Microbiology, Czech Academy of Sciences – Centre Algatech, Třeboň, Czechia

**Keywords:** type 4 secretion system (T4SS), biotin, Jekyll and Hyde, dinoflagellates, phytoplankton-bacteria interactions, Roseobacter group, B12 auxotrophy

## Abstract

The marine bacterium *Dinoroseobacter shibae* shows a Jekyll-and-Hyde behavior in co-culture with the dinoflagellate *Prorocentrum minimum*: In the initial symbiotic phase it provides the essential vitamins B12 (cobalamin) and B1 (thiamine) to the algae. In the later pathogenic phase it kills the dinoflagellate. The killing phenotype is determined by the 191 kb plasmid and can be conjugated into other Roseobacters. From a transposon-library of *D. shibae* we retrieved 28 mutants whose insertion sites were located on the 191 kb plasmid. We co-cultivated each of them with *P. minimum* in L1 medium lacking vitamin B12. With 20 mutant strains no algal growth beyond the axenic control lacking B12 occurred. Several of these genes were predicted to encode proteins from the type IV secretion system (T4SS). They are apparently essential for establishing the symbiosis. With five transposon mutant strains, the initial symbiotic phase was intact but the later pathogenic phase was lost in co-culture. In three of them the insertion sites were located in an operon predicted to encode genes for biotin (B7) uptake. Both *P. minimum* and *D. shibae* are auxotrophic for biotin. We hypothesize that the bacterium depletes the medium from biotin resulting in apoptosis of the dinoflagellate.

## Introduction

The phytoplankton of the world’s ocean is responsible for about 50% of the global net primary production (Field et al., 1998; Behrenfeld et al., 2001). Eukaryotic algae (e.g., diatoms, chlorophytes, and coccolithophores) are the most important contributors with about 90%, while only 10% is provided by cyanobacteria (Rousseaux and Gregg, 2013). About half of the marine algae species are dependent on vitamin B12 (cobalamin) for growth, while the other half use enzymes which do not require B12 as a co-factor (Croft et al., 2006; Tang et al., 2010). B12 is exclusively produced by certain Bacteria and Archaea (Croft et al., 2005). Because vitamin concentrations in the ocean water are low, algae are therefore dependent on symbiosis with bacteria (Croft et al., 2006; Smith et al., 2007). One bacterial group frequently associated with algae are the Roseobacters, which have recently been renamed *Roseobacteraceae*, and represent the marine family of the *Rhodobacterales*, consisting of more than 300 species (Pujalte et al., 2014; Simon et al., 2017; Hördt et al., 2020; Liang et al., 2021).

Roseobacters are mainly found in coastal waters, where they can make up 20% of the total bacterioplankton cells (Wagner-Döbler and Biebl, 2006; Moran et al., 2007; Giebel et al., 2011). Most of the cultivated Roseobacters possess large genomes, between 3.5 to 5 Mbp, which allows them to be metabolically flexible, especially during the initial phase of algal blooms where they are most abundant (Brinkhoff et al., 2008; Dang et al., 2008; Slightom and Buchan, 2009; Newton et al., 2010; Moran et al., 2012).

Roseobacters are frequently attached to particles and form close interactions with algae (Dang and Lovell, 2000; Geng and Belas, 2010; Salta et al., 2013; Ramanan et al., 2016; Li et al., 2019). Frequently these interactions are symbiotic, for example the *Sulfitobacter* strain SA11 provides ammonium and the growth hormone indole acetic acid to its host, the diatom *Pseudo-nitzschia multiseries*, in exchange for organic carbon and heme iron compounds (Amin et al., 2015; Hogle et al., 2017). Detailed analyses of the transcriptome and metabolome of the diatom *Thalassiosira pseudonana* and the Roseobacter *Ruegeria pomeroyi* in co-culture revealed the release of sulfonates by the diatom (Durham et al., 2017). The Roseobacter *Dinoroseobacter shibae* supplies the Chlorophyte *Ostreococcus tauri* with the vitamin B12 in exchange for organic carbon and biotin which are provided by the algae (Cooper et al., 2019).

These symbioses play a major role in global biogeochemical cycling, e.g., the fixation of carbon dioxide and the production of dimethylsulfide (Geng and Belas, 2010; Seymour et al., 2017).

In addition to symbiotic interactions, some Roseobacters form so-called Jekyll and Hyde interactions, consisting of a switch of the bacteria from a mutualistic to a pathogenic phase which was observed with dinoflagellates (Wang et al., 2014) and coccolithophores (Seyedsayamdost et al., 2011b). This behavior creates a new nutrient source for the bacteria during algal senescence (Seymour et al., 2017). For example, *Phaeobacter inhibens* promotes the growth of its host *Emiliania huxleyi* by the production of the growth hormone indole acetic acid, but ultimately kills it (Segev et al., 2016). The related bacterium *Phaeobacter gallaeciensis* produces specific compounds, so called Roseobacticides, to kill *E. huxleyi* (Seyedsayamdost et al., 2011a).

*Dinoroseobacter shibae* also shows a symbiotic and a pathogenic interaction with its interaction partner, the dinoflagellate *Prorocentrum minimum* (Wagner-Döbler et al., 2010). The bacteria initially supply the algae with the essential vitamin B12, but kill them later (Wang et al., 2014). The genetic modules required for the killing of the dinoflagellate *P. minimum* by *D. shibae* were analyzed, and it was shown that curing of the 191 kb sized plasmid caused loss of the killing phenotype (Wang et al., 2015). This so called “killer plasmid” was recently shown to be sufficient for killing of the dinoflagellate: It was conjugated into *Phaeobacter inhibens* which was acquired the ability to kill *P. minimum* (Tomasch et al., 2021).

The 191 kb plasmid (pDS191) is the largest plasmid of *D. shibae*. It shares a large syntenic region with the 126 kb plasmid (pDS126), the so-called sister plasmid; about 80% of the sequence is identical between the two plasmids (Wagner-Döbler et al., 2010). The sister plasmid is not able to kill the dinoflagellate when it is transferred into *P. inhibens* (Tomasch et al., 2021). The 191 kb plasmid contains 181 protein encoding genes, of which 146 are shared with its sister plasmid (Wagner-Döbler et al., 2010). The plasmid is required for anaerobic growth (Ebert et al., 2013). It also contains a type IV secretion system (T4SS) which mediates conjugation (Petersen et al., 2012; Ebert et al., 2013; Patzelt et al., 2016; Tomasch et al., 2021). The genes and structure of the T4SS are conserved between *D. shibae* and the Vir gene cluster of *Agrobacterium tumefaciens* (Petersen et al., 2013).

In this study we identified mutations in a range of plasmid-borne genes which affected the interactive phenotypes in various ways. To this end, we investigated the growth of *P. minimum* in co-culture with *D. shibae* strains carrying transposon insertions located on the 191 kb plasmid.

## Materials and Methods

### Bacterial Strains and Cultivation Conditions

The transposon mutant strains used in this study (Table 1) were selected from a transposon library of *D. shibae* generated by mariner transposon mutagenesis (Ebert et al., 2013). The insertion sites of the transposons were determined by arbitrary PCR (Ebert et al., 2013). *D. shibae* DSM 16493*^T^* wild type strain and all the transposon mutants were grown at 30°C and agitated using a shaker at 160 rpm in the dark in defined sea water medium (SWM) supplemented with 5 mM succinate (Supplementary Table 1). Bacterial growth was measured at OD_600_ in an Amershan Ultropsec 2100 pro or an automated Bioscreen C system.

**TABLE 1:** Transposon mutants of *D. shibae* tested in this study.

Locus tag	Protein length aa	Product	Log2 fold		B.-H. adjusted		Mean logCPM	Insertion site	Homologous gene in *D. shibae*	% protein identity	E-Value	Co-cultiva-tion pattern
			change		p-value							
				
			24 vs 18	30 vs 24	24 vs 18	30 vs 24						
Dshi_3633	547	Type 1 glutamine amidotransferase (GATase1)-like	−0.587	0.811	0.910	0.469	7.576	36154 (R)	unique	n.d.	n.d.	1
Dshi_3634	75	multispecies hypothetical protein Rhodobacterales	0.175	−1.108	1.000	0.325	6.376	37755 (F)	Dshi_3969	88.00	2.E-43	3
Dshi_3636	97	periplasmic signal peptide containing protein	−0.768	1.048	0.807	0.311	9.642	38437 (F)	Dshi_3971	64.58	5.E-41	1
Dshi_3637	117	virB family protein (conjugation/type IV secretion)	−0.005	−1.125	1.000	0.209	7.371	38437 (F)	Dshi_3972	99.15	2.E-85	1
Dshi_3639	97	VirB2	−0.602	0.103	0.979	1.000	3.361	38851 (F)	Dshi_3973	98.70	3.E-61	1
Dshi_3640	92	VirB3	−0.669	−0.204	0.963	0.978	4.463	40304 (F)	Dshi_3975	98.91	1.E-63	1
Dshi_3641	791	VirB4	−1.913	0.366	0.097	0.807	8.037	40435 (R)	Dshi_3976	94.78	0.E + 00	1
Dshi_3642	55	virB gene cluster (conjugation/type IV secretion)	4.887	0.888	0.956	0.734	1.345	42731 (R)	Dshi_3977	74.55	4.E-26	1
Dshi_3652	305	possible nuclease	0.652	0.043	0.859	1.000	8.016	51111 (F)	unique	n.d.	n.d.	2
Dshi_3653	516	DNA-directed DNA polymerase	0.247	0.106	0.979	0.982	8.087	52496 (R)	unique	n.d.	n.d.	1
Dshi_3654	566	possible retrotransposal reverse transcriptase	−0.584	0.257	0.818	0.842	9.019	54621 (F)	unique	n.d.	n.d.	1
Dshi_3666	54	possible transposase	0.735	−0.467	0.910	0.841	7.722	68387 (F)	unique	n.d.	n.d.	2
Dshi_3667	111	zinc-finger containing protein; possible transcription factor	−0.426	−0.460	0.930	0.737	8.523	68980 (R)	Dshi_2217	45.95	1.E-20	1
Dshi_3671	153	Uncharacterized protein YjbI containing pentapeptide repeats	8.877	−0.762	0.488	0.729	3.930	72004 (R)	Dshi_2635	30.43	2.E + 04	1
Dshi_3684	310	2OG-Fe(II) oxygenase superfamily domain containing protein	1.867	−0.563	0.039	0.576	10.655	89405 (F)	unique	n.d.	n.d.	3
Dshi_3685	189	biotin transporter BioY	1.163	−0.215	0.353	0.878	9.448	90384 (F)	Dshi_3688	36.94	4.E-16	3
Dshi_3686	221	ATPase	1.327	−0.910	0.270	0.250	9.584	90779 (R)	Dshi_0321	90.00	2.E-28	3
Dshi_3691	100	hypothetical protein conserved in Alphaproteobacteria	6.388	1.424	0.878	0.473	2.826	94661 (R)	Dshi_4000	89.00	2.E-58	1
Dshi_3693	128	hypothetical protein conserved in Alphaproteobacteria	−2.665	1.356	0.540	0.486	2.957	95574 (F)	Dshi_4002	93.75	3.E-89	1
Dshi_3701	399	putative transposase	−0.711	1.838	0.946	0.109	5.431	108585 (F)	unique	n.d.	n.d.	1
Dshi_3702	279	fatty acid hydroxylase	1.138	−0.605	0.488	0.525	8.272	109902 (R)	unique	n.d.	n.d.	3
Dshi_3713	158	hypothetical protein	1.624	1.530	0.850	0.206	5.137	118506 (F)	unique	n.d.	n.d.	2
Dshi_3714	197	3-octaprenyl-4-hydroxybenzoate carboxy-lyase	6.909	0.627	0.826	0.779	2.822	119328 (F)	unique	n.d.	n.d.	1
Dshi_3717	485	aldehyde dehydrogenase	−1.987	0.388	0.621	0.900	3.966	122468 (F)	Dshi_3021	97.00	2.E-73	1
Dshi_3718	305	AraC family transcriptional regulator	0.473	0.911	0.926	0.352	8.803	123896 (R)	Dshi_1525	34.28	2.E-48	1
Dshi_3722	511	AMP-dependent synthetase and ligase	−1.145	0.473	0.776	0.796	6.066	128996 (R)	Dshi_0700	31.03	2.E-54	1
Dshi_3730	108	putative regulator PrlF	1.455	0.965	0.590	0.513	8.697	137365 (R)	Dshi_4012	98.15	2.E-74	1
Dshi_3742	156	hypothetical protein	−1.293	1.257	0.913	0.592	3.378	148666 (F)	Dshi_4019	78.71	3.E-86	1

*Locus tag, protein length, and predicted gene product of the studied transposon mutants.*

*Log2 fold change and p-value of the genes during co-culture (Wang et al., 2015).*

*Insertion site of the transposon (Ebert et al., 2013).*

*Locus tag protein identity and E-value of the homologous gene.*

*Growth pattern found during co-cultivation of the transposon mutants.*

### Algal Strains and Cultivation Conditions

The axenic culture of *Prorocentrum minimum* strain CCMP 1329 used in this study was obtained from the Provasoli-Guillard National Center for Marine Algae and Microbiota (NCMA). *P. minimum* CCMP 1329 was cultivated in L_1_-Si medium containing biotin (B7), cobalamin (B12), thiamine (B1) (Supplementary Table 2) in 100 ml batches in 300 ml Erlenmeyer flasks at 22°C under a 12:12 h light-dark cycle with a light intensity of about 40 μmol photons m^–2^s^–1^. The algal culture was maintained in our lab by transferring 1% of the culture volume to fresh medium every 4 weeks. Lack of contaminating bacteria was checked by streaking aliquots on LB and MB plates.

### Co-cultivation of *Dinoroseobacter shibae* Strains With *Prorocentrum minimum*

For the co-cultivation experiments, bacteria and algae were prepared in the following way: Bacterial strains were grown on MB plates, transferred to a liquid preculture in 25 ml SWM and grown for approximately 24 h. The preculture was scaled up to 100 ml in SWM in a 300-ml flask with an initial OD_600_ of 0.03, grown to the late exponential phase (about 24 h of growth), washed once by centrifugation at 5,000 rpm for 5 min, and resuspended in L_1_-Si medium lacking vitamin B_12_ (L_1_-Si-B_12_). The culture of *P. minimum* was grown to the late exponential phase (about 14 days after routine transfer) on L1-Si medium to a density of ∼3 × 10^5^ cells/ml. The cell numbers of bacterial and algal pre-cultures were determined by flow cytometry as described previously and the inocula for co-cultivation experiments were adjusted accordingly (Wang et al., 2014). The co-culture was obtained by diluting the *P. minimum* culture to a final density of 2000 cells/ml in 100 ml fresh L_1_-Si-B_12_ medium for experiments in Erlenmeyer flasks and 10 ml for experiments in microtiter plates. The bacterial culture was added to a final density of 10^7^ cells/ml. For experiments in Erlenmeyer flasks, triplicates of 100 ml co-culture were followed. In microtiter plates eight replicate cultures of 200 μl were followed. The co-cultures and controls were incubated under the same conditions as the algal culture. The growth of *P. minimum* was examined according to the autofluorescence of its chlorophyll, which was measured using TECAN Infinite 200 microplate reader at λ_ex_ = 466 nm and λ_em_ = 678 nm (Johnsen et al., 1994).

## Results

### Selection of Transposon Mutants

**FIGURE 1:**
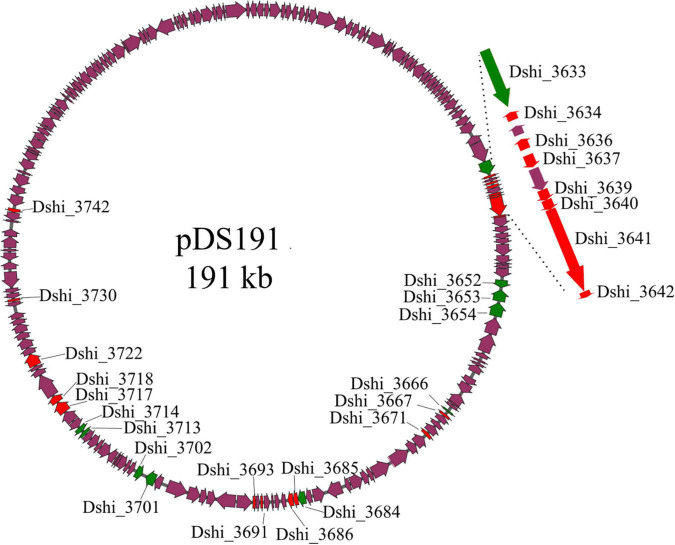
Localization of genes on plasmid p191 kb inactivated by transposon mutangenesis investigated in this study. Genes that are unique to the 191 kb plasmid are marked in green, genes that possess homologs on either the chromosome or the 126 kb plasmid are highlighted in red, purple genes were not studied or possessed no homologs. The virB gene cluster is shown enlarged.

In our previous study the transcriptome of *D. shibae* during co-culture with *P. minimum* had been analyzed (Wang et al., 2015). Supplementary Table 3 shows the expression of the genes on the 191 kb “killer plasmid.” Of the 184 protein-encoding genes on the 191 kb plasmid, 104 genes were highly expressed during co-culture, of which 4 showed significant differential expression when comparing algal growth phases. Here we selected 28 genes (Table 1), which were predicted to have interesting biological functions, and/or were unique to the 191 kb plasmid, or were highly expressed during all growth phases, and for which transposon mutants were available (Table 1). The location of these 28 genes on the 191 kb plasmid is shown in Figure 1, which also indicates if the gene was unique for the 191 kb plasmid or shared with the 126 kb sister plasmid or elsewhere on the chromosome. Dshi_3633 to Dshi_3642 are part of the type IV secretion system of *D. shibae*, which is present on both sister plasmids and is required for conjugation (Patzelt et al., 2016). Dshi_3642 was very highly differentially expressed after 24 days (logFC 24 vs 18 days: 4.89). Dshi_3652 to Dshi_3667 encoded proteins unique to the 191 kb plasmid, Dshi_3653 encoded a DNA-directed DNA polymerase, the remaining genes encoded hypothetical proteins. Dshi_3671 encoded a pentapeptide repeat-containing protein and was very highly expressed during exponential growth (logFC 24 vs 18 days: 8.88). Genes Dshi_3684 to Dshi_3686 were part of an operon related to uptake of biotin; they were not found on the 126 kb sister plasmid. A homolog to Dshi_3686 was present on the chromosome of *D. shibae*. A related protein to BioY (Dshi_3685) was found on the 191 kb plasmid (Dshi_3688). While Dshi_3685 was upregulated during exponential growth, Dshi_3688 was upregulated in the stationary growth phase of the algae. Two other genes highly expressed in the co-culture were Dshi_3691 and Dshi_3693, encoding hypothetical proteins, for which homologs were found on the sister plasmid. We also tested seven transposon mutants for genes encoding metabolic functions, which might be related to toxin synthesis, all of which were unique for the 191 kb plasmid, (Dshi_3701, Dshi_3702, Dshi_3713, Dshi_3714, Dshi_3717, Dshi_3718, Dshi_3722). Finally, we tested Dshi_3730 because of its potential regulatory role, and Dshi_3742 because it was relative highly expressed during stationary growth of the algae.

All the transposon mutants showed a delayed growth. While the wild-type reached stationary phase after 18 h, most mutants strains took up to 24 h. Three strains Dshi_3637, Dshi_3684 and Dshi_3714 took 30 h, but all strains reached the same final density (Supplementary Figure 1).

### Experimental Design

To study the effect of 28 transposon mutant strains on the growth of the dinoflagellate we had to scale down the co-cultivation experiment from the previous 100 ml Erlenmeyer flask volume to microtiter scale. In which, growth of the algae was determined by chlorophyll autofluorescence. A calibration experiment where algae growth was determined by flow cytometry and autofluorescence in parallel showed good correlation (Supplementary Figure 2). We then tested if the killer phenotype occurred also in microtiter scale similarly as in 100 ml Erlenmeyer flasks (Supplementary Figure 3) and confirmed that this was the case. The microtiter setup allowed us to investigate 12 different strains in parallel with eight replicas per strain.

### Growth of *P. minimum* in Co-culture

#### Three Types of Growth Patterns Could Be Distinguished

**FIGURE 2:**
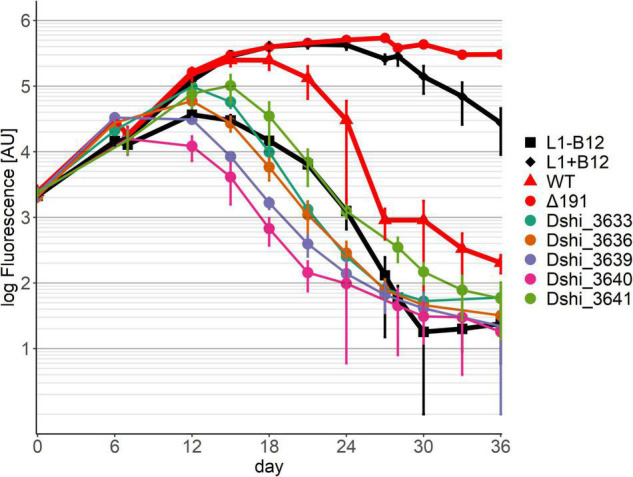
Co-cultivation pattern type 1 (symbiosis genes): No growth of the dinoflagellate in co-culture with the respective transposon mutant strains of *D. shibae*. Six examples are shown here. Growth of the dinoflagellate with the remaining 14 transposon mutants showing the same pattern can be found in Supplementary Figure 4. Controls are axenic cultures of *P. minimum* with/without B12, shown in black, and co-cultures with *D. shibae* wild-type and the Δ 191 plasmid mutant, shown in red.

In pattern 1, the growth of the algae was similar to growth in medium lacking B12 (Figure 2). Since *P. minimum* is dependent on B12 for growth, on this medium its growth was therefore massively impaired. Only a small increase of chlorophyll fluorescence was observed for about 6 days, most likely caused by the carry-over of vitamin B12 from the pre-culture. No further growth could be observed. Alga fluorescence declined and was always lower than in the co-culture with the wild-type, even during the pathogenic phase of growth where the wild-type killed the algae (after about 24 days). We conclude that the mutualistic interaction between dinoflagellate and bacterium could not be established. This pattern was observed for 20 transposon mutant strains (Table 1). Among them were all the transposon mutants for the type IV secretion system. Several genes unique to the 191 kb plasmid also exhibited pattern 1 behavior in co-culture: a DNA-directed DNA polymerase (Dshi_3653), a transposase (Dshi_3701); three genes encoding metabolic enzymes: 3-octyprenyl-4-hydroxybenzoate carboxy-lyase (Dshi_3714), an aldehyde dehydrogenase (Dshi_3717), and an AMP-dependent synthetase and ligase (Dshi_3722), and an AraC family transcriptional regulator (Dshi_3718).

**FIGURE 3:**
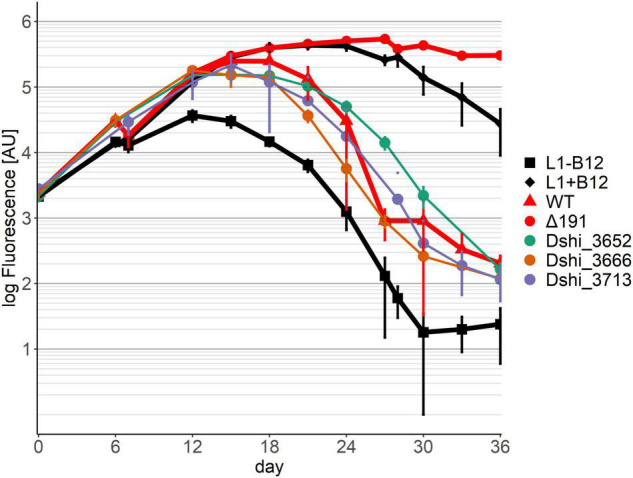
Co-cultivation pattern type 2 (neutral genes): The growth of the Dinoflagellate with the respective transposon mutant strain was the same as with the wild-type of *D. shibae*. Neither killing nor symbiosis were affected by these genes. Controls as in Figure 2.

In pattern 2 (Figure 3) the growth of the dinoflagellate with three transposon mutants had the same mutualistic phase, followed by a pathogenic phase as with the wild-type of *D. shibae*. Gene Dshi_3652 encoded a putative nuclease, Dshi_3666 a putative transposase and Dshi_3713 a hypothetical protein. We conclude that these genes have no effect on the interaction between the dinoflagellate and the bacterium since both the mutualistic and the pathogenic phase were fully present.

**FIGURE 4:**
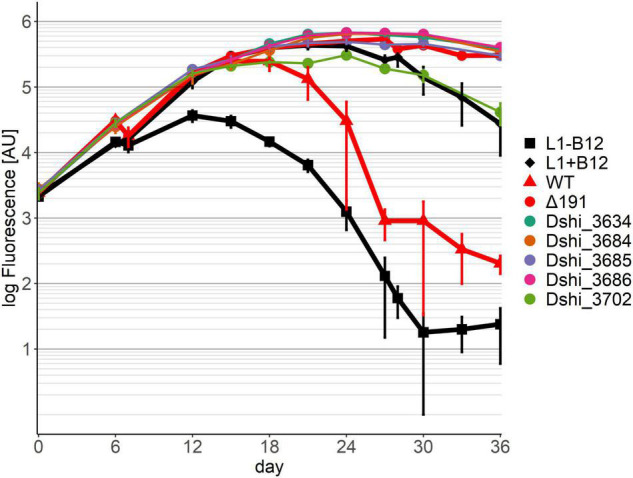
Co-cultivation pattern type 3 (killer genes): No killing of the dinoflagellate in co-culture with the respective transposon mutant strain of *D. shibae* was observed. Controls as in Figure 2.

Five transposon mutants were no longer able to kill the dinoflagellate (pattern 3) (Figure 4). With one of these mutants, Dshi_3702, which had a fatty acid hydroxylase kinactivated, the algae grew similar to an axenic culture supplemented with B12, but worse than in a co-culture with *D. shibae* lacking the entire 191 kb plasmid (Δ191 in Figure 4; Wang et al., 2015). Thus, there was no probiotic effect of this bacterial strain, although it was apparently able to provide B12 to the algae. The other four mutants showed growth similar to the *D. shibae* strain lacking the entire 191 kb plasmid. In transposon mutant Dshi_3634 a small 75 amino acid hypothetical protein is knocked out. Three transposon mutations (Dshi_3684, Dshi_3685 and Dshi_3686) were in genes that are organized in an operon. While Dshi_3684 is a hypothetical protein, Dshi_3685 encodes a member of the bioY protein family and Dshi_3686 is a substrate binding protein, which is part of an ABC transporter. The BioY proteins, encoding a high-affinity substrate-binding protein that interacts with the ABC transport protein, are involved in the uptake of biotin (Hebbeln et al., 2007; Finkenwirth et al., 2013).

## Discussion

In this study we examined the effect of transposon mutants of genes on the “killer plasmid” of *D. shibae* on the co-culture with *P. minimum* in L1 medium lacking vitamin B12 to identify genes required for symbiosis (e.g., supply of vitamin B12) or the killing of the dinoflagellate. Unexpectedly, we found that the majority of the inactivated genes (20 out of 28) were actually required to allow growth of the dinoflagellate beyond an axenic control that lacked B12 in the culture medium. Thus, these genes were necessary to provide B12 to the dinoflagellate. The 191 kb plasmid is therefore not only a killer plasmid, but also a symbiosis plasmid.

Among them several genes belonged to the T4SS of *D. shibae*. T4SS are bacterial nanomachines that can transport DNA into other bacteria *via* conjugation, and they are also the only known biological mechanism by which DNA can be introduced into Eukaryotes (Lacroix and Citovsky, 2016). They have evolved to deliver proteins and effector molecules into eukaryotes and thus play a large role for pathogenesis (Odenbreit et al., 2000).

In our study T4SSs that were made dysfunctional by transposon insertions did not allow growth of the dinoflagellate beyond that of an axenic control in medium lacking vitamin B12. Part of the T4SS are pili, in *Agrobacterium* encoded by VirB5 and VirB2 (Souza et al., 2015; Lacroix and Citovsky, 2016). These pili might be required to establish a close spatial relationship between algae and bacterium to make exchange of nutrients between them more efficient. However, the T4SS might also be fully functional and deliver DNA, proteins or small molecules to the algae. The T4SS of *D. shibae* was already shown to deliver DNA to other bacteria (Patzelt et al., 2016). Just like the virB gene cluster of *Agrobacterium*, it might also be able to deliver DNA into Eukaryotes. It is not possible to predict the function of a T4SS from its sequence (Odenbreit et al., 2000). These nanomachines might also transfer B12, precursors of it, or protein-vitamin complexes into the algae *via* the T4SS. Interestingly, an almost identical T4SS is located on the 126 kb plasmid. It is possible that the two T4SS have different functions.

We found only five genes that were necessary to kill the dinoflagellate. Three of them were organized in an operon predicted to be involved in the uptake of biotin. Apart from the three tested transposon mutants (Dshi_3684, Dshi_3685, Dshi_3686) this operon also contains the gene Dshi_3687 and Dshi_3683, for which no transposon mutants was available. Dshi_3687 is annotated as a cobalt/nickel transport protein, EcfT, which is a conserved part of uptake systems (Rodionov et al., 2006; Siche et al., 2010). Dshi_3683 is a resolvase domain containing protein. It is likely that the complete operon encodes an energy-coupling factor (ECF) ABC-transporter complex. These transporters consist of an ATPase (Dshi_3686), a conserved transmembrane protein (Dshi_3687) as well as a transmembrane substrate-capture protein (Dshi_3685) (Hebbeln et al., 2007). These high-affinity biotin uptake systems were studied in *Rhodobacter capsulatus* but their role in algae bacteria interactions is not known (Hebbeln et al., 2007). Dshi_3688 which is related to Dshi_3685 was not part of this operon, because it is located on the opposite strand, but would be an interesting gene for further investigation. This biotin uptake operon is unique for the 191 kb plasmid, which explains why killing of the dinoflagellate is mediated by the 191 kb plasmid, but not by the 126 kb sister plasmid (Tomasch et al., 2021).

Biotin is an essential vitamin for all living cells, both prokaryotes and eukaryotes. It is a cofactor for carboxylases enzymes which carry out central functions in the cell (Tong, 2013). The biosynthesis of biotin requires six enzymatic steps in *E. coli* (Streit and Entcheva, 2003). Most groups of algae (e.g., chlorophyta, haptophyte, diatoms) are able to produce biotin by themselves. Curiously, only dinophyta, and particularly bloom forming dinoflagellates, are auxotrophic for biotin, one of them being *P. minimum* (Croft et al., 2006; Tang et al., 2010).

Since dissolved biotin levels are low in the oceans and limiting for algae growth, this indicates that bloom forming dinoflagellates are crucially dependent on symbiosis with bacteria to supply them with biotin (Sanudo-Wilhelmy et al., 2012). They have a broad choice for the bacterial symbiont, since most bacteria are able to synthetize biotin (Streit and Entcheva, 2003; Cooper et al., 2019). In the Roseobacter group, however, all 52 genomes across the whole lineage analyzed carry B12 synthesis genes in their genomes, but only 22 genomes have the genes for biotin synthesis (Luo and Moran, 2014). *D. shibae* and several of the more ancient Roseobacters are auxotrophic for biotin, while *Phaeobacter* and related genera at the other end of the phylogenetic tree can synthesize it (Luo and Moran, 2014). Biotin synthesis genes were also not found in the recently cultivated lineage CUAB that has a streamlined genome (Feng et al., 2021). Accordingly, with respect to biotin, two types of symbioses are possible between algae and Roseobacters in nature, namely between auxotrophic algae (e.g., dinoflagellates) and a certain group of biotin producing Roseobacters, while algae that can synthesize biotin (chlorophyta, haptophytes, diatoms) can form symbioses with the other group of Roseobacters that cannot produce biotin. The interaction between *Ostreococcus tauri* and *D. shibae* is an example for this type of symbiosis (Cooper et al., 2019). Some Roseobacters (e.g., *Phaeobacter*) can synthesize all three vitamins that are present in L1 medium, (B12, thiamin, and biotin) and thus represent ideal probiotica for notorious to cultivate marine algae. In our model system, however, both *P. minimum* and *D. shibae* are dependent on the biotin supplied by the cultivation medium. Their interaction could only work in the Erlenmeyer flask regularly provided with L1 medium, but not in nature, except under the unlikely conditions of excess biotin in the environment. We hypothesize that *D. shibae* depletes the medium of biotin and in such a way causes biotin deficiency of the dinoflagellate which leads to apoptosis. Mutants of the model plant *Arabidopsis thaliana* unable to produce biotin showed spontaneous cell death (Li et al., 2012). In algae, programmed cell death can be triggered by nutrient starvation (Bidle, 2015).

To conclude, both symbiosis and killing are apparently mediated by the need for essential vitamins. In the case of B12, the bacteria provide it to the algae, resulting in a mutualistic symbiosis. In the case of biotin, algae and bacteria compete, and depletion of biotin from the medium by the highly efficient bacterial uptake system causes algal death. Our study points to the importance of the T4SS and a number of additional unique genes on the 191 kb plasmid for the delivery of B12 to *P. minimum*.

## Data Availability Statement

The original contributions presented in the study are included in the article/Supplementary Material, further inquiries can be directed to the corresponding author/s.

## Author Contributions

JM and IW-D wrote the manuscript. HW and JT performed the experiments and analyzed the data. ME, EH, and DJ provided and verified transposon mutants. IW-D designed the study. All authors contributed to the article and approved the submitted version.

## Conflict of Interest

The authors declare that the research was conducted in the absence of any commercial or financial relationships that could be construed as a potential conflict of interest.

## Publisher’s Note

All claims expressed in this article are solely those of the authors and do not necessarily represent those of their affiliated organizations, or those of the publisher, the editors and the reviewers. Any product that may be evaluated in this article, or claim that may be made by its manufacturer, is not guaranteed or endorsed by the publisher.

## Supplementary Material

Supplementary Figure 1 Growth of *D. shibae* transposon mutants. Bacteria were grown in SWM in microtiter plates and growth was determined by measuring OD_600_ using a Bioscreen instrument.

Supplementary Figure 2 Correlation flow cytometry—fluorescence. An axenic culture of *P. minimum* was sampled after 7 and 21 days, diluted with L1-Si with the following ratios 9:1, 8:2, 1:1, 1:4, 1:9. Algal density was measured by chlorophyll autofluorescence as well as flow cytometry.

Supplementary Figure 3 Comparison Erlenmeyer flask—microtiter plate. A co-culture between *P. minimum* and *D. shibae* wild-type was started in an Erlenmeyer flask (100 ml per culture) and a microtiter well plate (200 μl per culture) in parallel and followed for 36 days.

Supplementary Figure 4 Co-cultivation pattern 1 remaining strains: No growth of the dinoflagellate beyond the axenic control lacking B12 in co-culture with the respective transposon mutant strain of *D. shibae*.
