# Supplementary figures and images for: The Influence of Genes on the “Killer Plasmid” of Dinoroseobacter shibae on Its Symbiosis With the Dinoflagellate Prorocentrum minimum
Source: Front Microbiol. 2022 Jan 28;12:804767. doi: 10.3389/fmicb.2021.804767 (PMC8831719; doi:10.3389/fmicb.2021.804767)

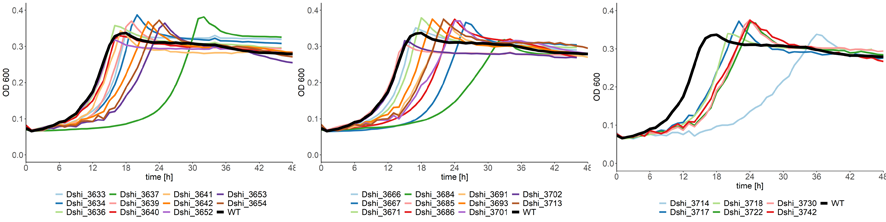

Supplement: Supplementary Figure 1 — Growth of D. shibae transposon mutants. Bacteria were grown in SWM in microtiter plates and growth was determined by measuring OD600 using a Bioscreen instrument. [file Image_1.PNG]

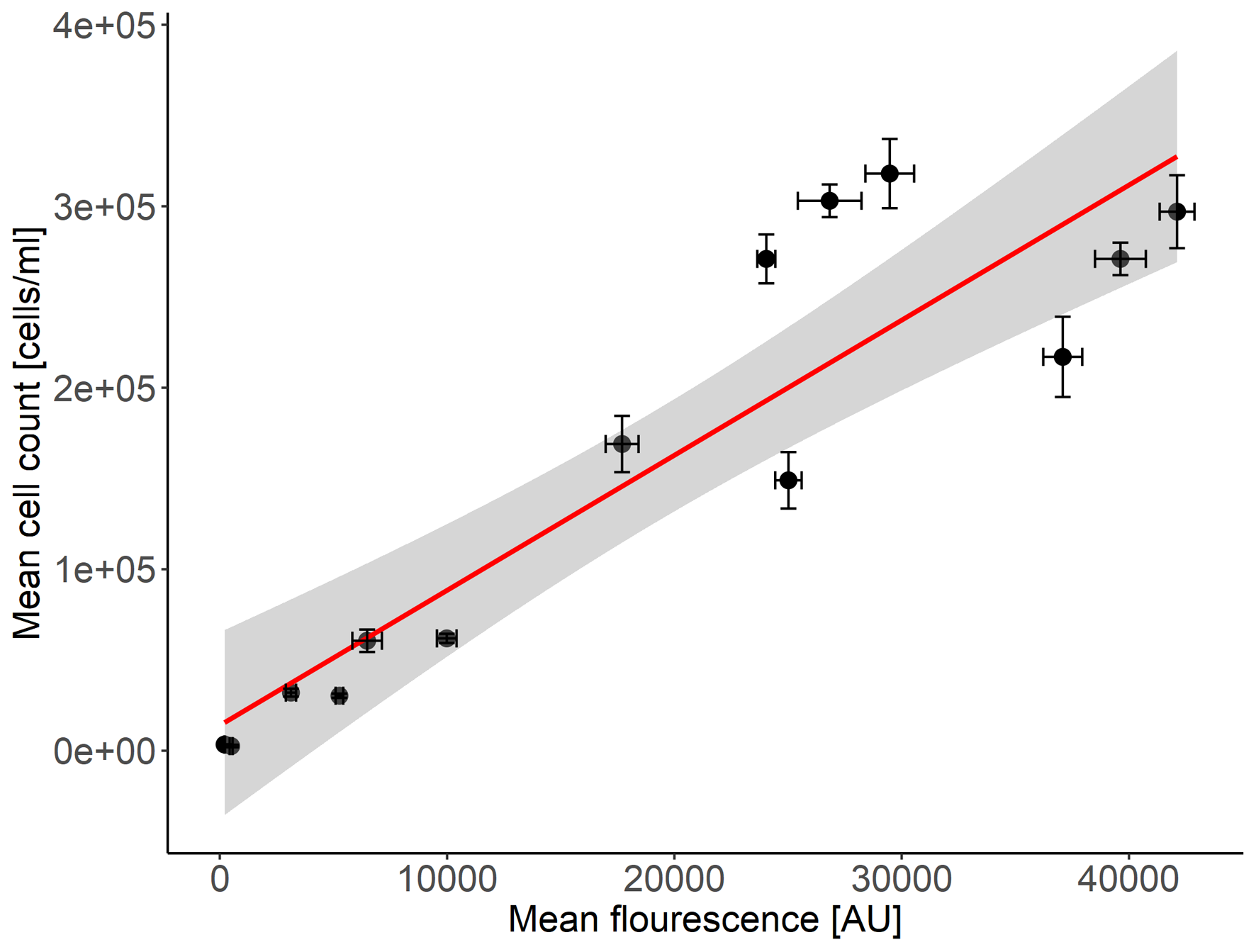

Supplement: Supplementary Figure 2 — Correlation flow cytometry—fluorescence. An axenic culture of P. minimum was sampled after 7 and 21 days, diluted with L1-Si with the following ratios 9:1, 8:2, 1:1, 1:4, 1:9. Algal density was measured by chlorophyll autofluorescence as well as flow cytometry. [file Image_2.PNG]

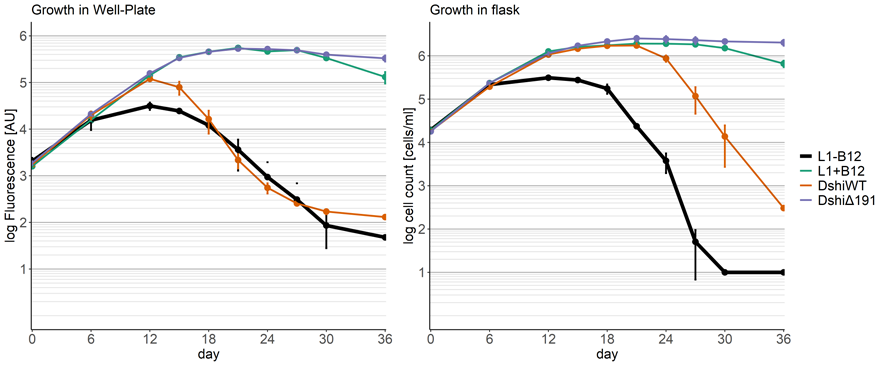

Supplement: Supplementary Figure 3 — Comparison Erlenmeyer flask—microtiter plate. A co-culture between P. minimum and D. shibae wild-type was started in an Erlenmeyer flask (100 ml per culture) and a microtiter well plate (200 μl per culture) in parallel and followed for 36 days. [file Image_3.PNG]

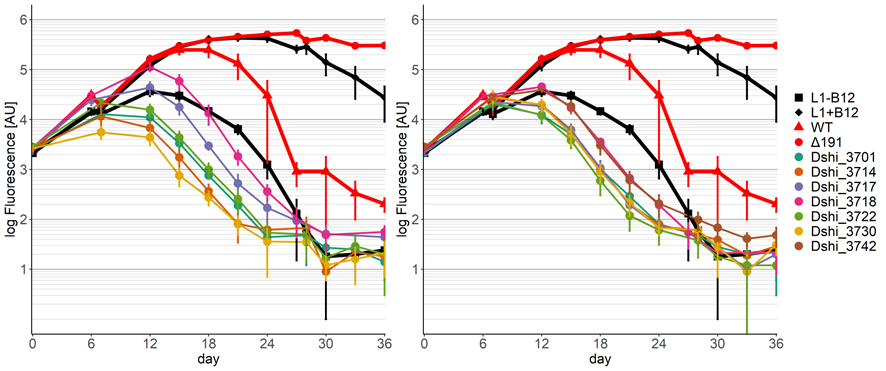

Supplement: Supplementary Figure 4 — Co-cultivation pattern 1 remaining strains: No growth of the dinoflagellate beyond the axenic control lacking B12 in co-culture with the respective transposon mutant strain of D. shibae. [file Image_4.PNG]
